# Influence of the Hydrophobicity of Pluronic Micelles Encapsulating Curcumin on the Membrane Permeability and Enhancement of Photoinduced Antibacterial Activity

**DOI:** 10.3390/pharmaceutics14102137

**Published:** 2022-10-08

**Authors:** Maria Antonia Tănase, Andreia Cristina Soare, Lia Mara Diţu, Cristina Lavinia Nistor, Catalin Ionut Mihaescu, Ioana Catalina Gifu, Cristian Petcu, Ludmila Otilia Cinteza

**Affiliations:** 1 Physical Chemistry Department, University of Bucharest, 030018 Bucharest, Romania; 2 Microbiology Department, Faculty of Biology, University of Bucharest, 60101 Bucharest, Romania; 3 Polymer Department, National Institute for Research and Development in Chemistry and Petrochemistry-ICECHIM, 202 Spl. Independentei, 060021 Bucharest, Romania

**Keywords:** polymeric micelles, curcumin, microbial photoinactivation, photodynamic therapy, membrane permeabilization

## Abstract

Apart from its well-known activity as an antimicrobial agent, Curcumin (CURC) has recently started to arouse interest as a photosensitizer in the photodynamic therapy of bacterial infections. The aim of the present study was to evidence the influence of the encapsulation of Curcumin into polymeric micelles on the efficiency of photoinduced microbial inhibition. The influence of the hydrophobicity of the selected Pluronics (P84, P123, and F127) on the encapsulation, stability, and antimicrobial efficiency of CURC-loaded micelles was investigated. In addition, the size, morphology, and drug-loading capacity of the micellar drug delivery systems have been characterized. The influence of the presence of micellar aggregates and unassociated molecules of various Pluronics on the membrane permeability was investigated on both normal and resistant microbial strains of *E. coli*, *S. aureus*, and *C. albicans*. The antimicrobial efficiency on the common pathogens was assessed for CURC-loaded polymeric micelles in dark conditions and activated by blue laser light (470 nm). Significant results in the reduction of the microorganism’s growth were found in cultures of *C. albicans*, even at very low concentrations of surfactants and Curcumin. Unlike the membrane permeabilization effect of the monomeric solution of Pluronics, reported in the case of tumoral cells, a limited permeabilization effect was found on the studied microorganisms. Encapsulation of the Curcumin in Pluronic P84 and P123 at very low, nontoxic concentrations for photosensitizer and drug-carrier, produced CURC-loaded micelles that prove to be effective in the light-activated inhibition of resistant species of Gram-positive bacteria and fungi.

## 1. Introduction

Curcumin ([1,7-bis(4-hydroxy-3-methoxyphenyl)-1,6-heptadiene-3,5-dione]) is the major polyphenolic compound belonging to the curcuminoid family extracted from the dried rhizome of the herb *Curcuma Longa* (also known as turmenic), together with demethoxycurcumin and bisdemethoxycurcumin. Curcumin (CURC) has been found to be a useful tool in a variety of diseases, from cardiovascular diseases [1] to cancer [2] and COVID-19 infection [3].

Another major therapeutic use of CURC is based on its antibacterial activity against a large variety of microorganisms ranging from Gram-positive and Gram-negative bacteria to fungi [4].

Due to its photophysical properties, Curcumin was found to be effective in photodynamic therapy (PDT) against tumoral cells or the photoinduced inactivation of microorganisms. There is a large variety of studies in which CURC was successfully used as photosensitizer (PS) against different pathogens [5] or tumoral cells [6].

The main drawback of CURC is its highly hydrophobic character (logP = 4.12) that results in a poor water solubility, reduced bioavailability, and rapid metabolism. Thus, the enhancement of the physical-chemical and pharmacological properties of CURC is a crucial issue for its clinical application. In the context of PDT applications, it is important to keep in mind that the vehicle must preserve the properties of the photosensitizers, in order to avoid the loss or modification of their activity on microorganisms.

The encapsulation of CURC in various drug delivery systems was performed using colloidal systems such as liposomes [7], micelles [8], hydrogels and polymeric scaffolds, microemulsions [9], gel microemulsions [10], or nanoparticles [11]. Among these nanostructured carriers proposed for the delivery of drugs, the polymeric micelles (PM) have found their place as an effective solution, in particular for poorly water-soluble compounds.

Extensive attention has been paid to the class of copolymers called Poloxamers (trade name Pluronics^®^), amphiphilic in nature with surfactant properties, which contain in their structure poly(ethylene oxide) (PEO) and poly(propylene oxide) (PPO) blocks, disposed in a PEO–PPO–PEO triblock structure. Depending on the number of poly(ethylene oxide) and poly (propylene oxide) units, Pluronics^®^ exhibit different physicochemical properties, including Hydrophilic Lipophilic Balance (HLB) and self-assembling behavior (critical micellar concentration). Among their conveniences are their low toxicity and increased bioavailability, some of them (F127, F68, F88) being FDA approved as pharmaceutical excipients [12]. Pluronics^®^ micelles have been extensively studied as carriers for many antitumoral drugs [13] as well as for antimicrobial substances [14]. Pluronic polymers were also found to increase the bioavailability of various antibacterial drugs and, consequently, produce the enhancement of their activity [15].

Pluronics^®^ micelles were also investigated as carriers for Curcumin, and the effect of the drug’s encapsulation on the antitumoral, antioxidant, and antibacterial activity was evidenced. For example, CURC loaded in Pluronic^®^ F127 or P123 micelles exhibits enhanced solubility, and the formulation induced enhanced cytotoxic effects in human breast carcinoma [16]. Curcumin-loaded Pluronic^®^ F-127 micelles have been evaluated as photosensitizers in PDT for oral applications against *Streptococcus mutans* and *Candida albicans* biofilms [17]. The photoinhibitory activity of CURC encapsulated in Pluronic^®^ P-123 micelles was reported in a study conducted by Dias et al. [18], and the influence of the pH on the growth reduction of *Staphylococcus aureus* strains was evidenced.

It is currently accepted that an important mechanism of antimicrobial resistance is the overexpression of efflux pumps; thus, great efforts have been made to develop methods to inhibit these membrane transporters responsible for expelling out antibiotics from the microbial cell, and thus, various peptides and other small molecules were tested [19]. A very peculiar property of the Pluronics^®^ derivatives is their ability to induce multidrug resistance reversal in tumoral cells, through multiple mechanisms. However, very little research was performed on the influence of Pluronics on the membrane fluidization and multidrug resistance reversal in microbial cells [20].

The aim of the present work was to investigate the influence of the encapsulation of Curcumin into Pluronic micelles on the efficiency in light-driven antibacterial growth reduction. The molecular composition and hydrophobicity of triblock copolymers was studied, regarding the efficiency of drug encapsulation and also the interaction with bacterial and yeast cells. Three polymer derivatives were investigated, with various degree of hydrophobicity and various length of PPO and PEO segments, targeting the development of a drug delivery system with a low content of surfactant and enhanced stability of encapsulated CURC. We presumed that the presence of unassociated Pluronic molecules and micellar aggregates could exert a certain effect on the membrane’s permeability, and the changes in CURC photosensitization activity were evidenced. CURC is considered a safe active ingredient, since it is reported to have no systemic toxic effect on human health even at high doses of up to 8 g/day [21]. However, some recent papers report side effecst for normal cells at medium and high concentrations [22,23], and we explored the possibility of obtaining significant efficiency in pathogen photoinactivation with a reduced amount of CURC as the photosensitizer.

## 2. Materials and Methods

### 2.1. Materials

Curcumin (MW = 368.38), Pluronic^®^ F127, Pluronic^®^ P84 and Pluronic^®^ P123, Pyrene, *N*-phenyl-1-naphthylamine (NPN) and propidium iodide (PI) aqueous solution 1 mg/mL were purchased from Sigma Aldrich (Merck Group, Darmstadt, Germany). Solvents used, dimethysulfoxide (DMSO) and ethanol, were obtained from Honeywell (Charlotte, CA, USA), and Phosphate Buffered Saline 10× (PBS) from Remed Prodimex (Bucharest, Romania). Distilled water was produced in our laboratory. All chemical and reagents were used without any purification or adjustments.

### 2.2. Methods

#### 2.2.1. Micelles Preparation

Three Pluronic derivatives, Pluronic^®^ F127, Pluronic^®^ P84, and Pluronic^®^ P123, with different molecular weights and lengths of their PEO and PPO blocks, were employed to produce micellar dispersions. Pluronic micellar solutions of 1.5% (*w*/*v*) in distilled water or PBS were prepared using the direct dissolution method, since it is reported as the easiest and most convenient method for block copolymer micelles fabrication [24]. Briefly, the required amount of polymer was dissolved in water or PBS to obtain the desired concentrations, and the solution was left to equilibrate for 12 h under magnetic stirring.

For drug-loaded micelles, CURC was encapsulated by the thin film hydration. Curcumin was dissolved in ethanol to obtain a stock solution with the concentration of 1 mg/mL. Specific volumes were transferred in a round bottom flask, which was attached to a rotary evaporator (Rottavapor-R-300^®^, Buchi Labortechnik AG, Flawil, Switzerland) and heated at 50 °C under vacuum to assure a thin, evenly distributed film of curcumin remanent on the walls. After all the solvent was removed, the required volume of Pluronic micellar solution or other solvents was added to rehydrate the film under constant low rate stirring (less than 100 rpm) for about an hour. The as-prepared systems with Curcumin were filtered through the sterile syringe filter Minisart^®^ 0.2 μm (Sartorius, Gottingen, Germany) and used for further experiments.

#### 2.2.2. Characterization of Micelles

The CMC of Pluronic^®^ F127, Pluronic^®^ P84, and Pluronic^®^ P123 was determined using a fluorescence assay with pyrene as a fluorescent probe [25]. A stock solution of pyrene (1 × 10^−6^ M) was obtained by dissolving the required amount of pyrene in ethanol. Then, the solvent evaporated under normal pressure to produce a thin film on the vial wall. The film obtained was rehydrated in distilled water under magnetic stirring, in dark conditions, for 24 h. Polymeric solutions of various concentrations were prepared by successive dilutions of Pluronic derivatives using the pyrene stock solution described earlier.

All spectrofluorimetric measurements were achieved using a Jasco FP6300 spectrofluorimeter (Jasco Corporation, Tokyo, Japan). The excitation spectra of pyrene in Pluronic solutions were recorded as being between 300–370 nm, with the emission wavelength set at 390 nm. The slits were fixed at 2.5 nm for both excitation and emission. The slope modification in the graphical representations of the ratio between the intensities I1 (the peak at 334 nm) to I3 (the peak at 337 nm) against the log of the micelle concentration indicates the CMC value [26].

Pure and Curcumin-loaded micellar systems in PBS were evaluated for their particle size, polydispersity index, and Zeta potential using the Dynamic Light Scattering (DLS) method. Experiments were performed at 37 °C without any further dilution of the samples. The stability of the above-mentioned systems was assessed by measuring the variation in the size and size distributions of the samples after 4 weeks. All experiments were performed using a Nano ZS Zetasizer (Malvern Instruments Ltd., Malvern, UK), with a He-Ne laser 532 nm and scattering angle of 173°. The average diameter of the micelles and the polydispersity index (PDI) were calculated using the software of the instrument. The data reported were mean values obtained from 3–5 measurements for each sample, in automatic mode.

The morphology of the void and loaded polymeric micelles was investigated by transmission electron microscopy (TEM). The samples were prepared from diluted samples (ten folds) in distilled water, without prior filtration. One to two drops of micellar solution were deposited on the carbon Lacey Formvar/Carbon film, copper grids (Ted Pella Inc., Redding, CA, USA), the excess was removed using a filter paper, and the sample was left to dry. The images were obtained using a TECNAI F20 G² TWIN Cryo-TEM instrument (FEI Company, Amsterdam, The Netherlands) operating at an accelerating voltage of 200 kV.

Curcumin interaction with Pluronic micellar systems was investigated using Fourier Transformed Infrared Spectrometry (FTIR). Preparation of the sample pellets implied adding Curcumin-loaded Pluronic micellar solutions to KBr powder, followed by a drying procedure, at low temperatures in a vacuum, for solvent removal. Curcumin powder and Pluronic dried samples mixed in KBr powder were used to prepare reference pellets. All spectra were recorded in the 4000–400 cm^−1^ spectral domain, with 32 scans at a resolution of 4 cm^−1^ using a Tensor 37 Bruker system (Woodstock, NY, USA).

#### 2.2.3. Optical Properties of Curcumin-Loaded Polymeric Micelles

Absorption and fluorescence spectra of Curcumin solubilized in Pluronic micelles and in various model solvents were recorded. Curcumin was dissolved in ethanol, DMSO, acetonitrile, and 1.5% (*w*/*v*) Pluronic solutions in PBS to obtain solutions with the final concentration of 0.005 mg/mL. The measurements were carried out by using a Jasco V-660 spectrometer (Jasco Corporation, Tokyo, Japan) and a Jasco FP6300 spectrofluorimeter (Jasco Corporation, Tokyo, Japan).

The photostability of the drug encapsulated in micelles was investigated using changes in fluorescence emissions after exposure to blue light. Curcumin in different Pluronic micellar solutions (0.2 μg/mL) were prepared as described earlier. Samples were placed in a quartz cuvette (1 cm optical pathway) and excited at 470 nm using the optical system of the above-mentioned spectrofluorimeter for 5, 15, 30, and 45 min, and the fluorescence spectra were recorded. The amount of undecayed CURC was calculated using the equation:

(1) $$Undecayed\;Curcumin\%=\frac{Amount\;of\;Curcumin\;Determined}{Amount\;of\;Curcumin\;Used}\times 100$$

#### 2.2.4. Drug Solubility and Entrapment Efficiency

Solubility of Curcumin in Pluronic micelles was evaluated based on the fluorimetric method for the quantification of drugs, adapted in our laboratory from the literature [27]. Fluorescence spectra were recorded in ethanolic solutions with λ_ex_ = 425 nm and an emission wavelength in the range of 450–700 nm. The maximum emission at 525 nm was selected to be plotted versus the Curcumin concentration.

Calibration curves of CURC in ethanol with or without the presence of Pluronics (0.15 mg/mL) were constructed, and in all cases, linearity was observed in the domain 0.05–0.5 μg/mL.

The solubilization samples were prepared by adding a suitable volume of Curcumin stock solution in ethanol in a vial, and the solvent was slowly evaporated. Specific amounts of Pluronics 1.5% (*w*/*v*) micellar solution were added to obtain solutions with a Curcumin:Pluronic ratio of 1:20. Drug–polymer systems were left to equilibrate overnight at room temperature, in the dark, and then were filtered using a Minisart^®^ 0.2 μm seringe filter. The samples were analyzed by fluorescence and quantified using the calibration curves obtained before.

Encapsulation efficiency (EE%) and drug loading (DL%) were calculated according to the equations:

(2) $$EE\%=\frac{Amount\;of\;Curcumin\;Encapsulated}{Amount\;of\;Curcumin\;Used}\times 100$$

and

(3) $$DL\%=\frac{Amount\;of\;loaded\;Curcumin\;}{Amount\;of\;Polymeric\;Micelles}\times 100$$

#### 2.2.5. In Vitro Drug Release

The release kinetics of CURC from Pluronic micelles was studied using the dialysis bag method, using a water–ethanol solution as the receptor fluid. A volume of 3 mL of Pluronic micelles loaded with Curcumin (0.037 mg/mL) was introduced to a pre-swelled dialysis bag. Each bag was placed in a 500 mL volumetric flask containing 50/50 (*v*/*v*) ethanol: water release medium at 37 °C, in the dark, under constant stirring at less than 100 rpm. A volume of 2 mL was withdrawn from the release medium at established time intervals and replaced with the same amount of freshly prepared release medium. Samples were quantified by fluorescence spectrometry under the same conditions as the ones described in the previous section.

#### 2.2.6. Microbial Membrane Permeability Assessment

The microbial cell membrane permeability was investigated using 1-*N*-phenylnapthylamine (NPN) and Propidium Iodide (PI) as fluorescent probes. The samples analyzed were bacterial and fungi cultures incubated with Pluronic solutions at different concentrations, Curcumin in DMSO and Curcumin in Pluronic micelles. The composition of the tested systems is presented in Table 1.

Microbial suspensions and sample preparations were performed by using a protocol developed in our laboratory, adapted from the literature’s recommendations [28]. Bacterial suspensions obtained as described in Section 2.2.6. were diluted to OD600 = 0.1 and further used in all the experiments. In order to evaluate the influence of the Pluronic solutions and CURC-loaded Pluronic micelles on the membrane’s permeability, 1 mL of each sample was diluted 1:10 with microbial suspension and incubated for 2 h at 37 °C. Control samples with PBS, DMSO, and CURC in DMSO were prepared in the same manner. After the incubation time, volumes of 2 mL of microbial culture exposed to tested solutions were placed in Eppendorf tubes and then subjected to centrifugation at 11000 rpm for 10 min in order to obtain cell pellets. The supernatant was removed, and the cells were washed in PBS, repeating the procedure three times. A volume of 1 mL of resuspended cells was mixed with an appropriate volume of NPN and PI stock solutions in order to obtain a final concentration of 20 μM for NPN and 7.5 μM for PI, respectively. The stock solution of NPN was prepared by dissolving the fluorescent dye in acetone at a concentration of 10 mM. The as-prepared cell dispersion with dyes was incubated in dark conditions for 10 min and then transferred in a black fluorimeter plate well. The uptake of the fluorescent probe NPN was evaluated by measuring the fluorescence emissions at 460 nm when excited with a 360 nm lamp. The conditions for PI uptake determination are excitation at 540 nm and emission at 620, respectively. The plates were read in automatic mode using a Synergy HTX Multi-Mode Microplate Reader (BioTek Instruments, Rochester, VT, USA).

The increase in the fluorescence emissions of NPN is considered as evidence of the outer membrane’s integrity damage, while the increase in the fluorescence emission of PI evidence the fluidization of the cell membrane. The relative fluorescence intensity was expressed relative to the control sample, with an untreated microbial strain considered equal to a unit. All the data presented are the mean ± SD of three independent experiments.

#### 2.2.7. Antimicrobial Activity and Photoinactivation

The antimicrobial assay was performed using both standard and clinical microbial strains that are included in the microbial collection of the University of Bucharest, Faculty of Biology, Microbiology Department: *Staphylococcus aureus* ATCC 25923 and *Staphylococcus aureus* MRSA 5579 (clinical isolate)*, Escherichia coli* ATCC 25922 and *Escherichia coli* ESBL 135 (clinical isolate), *Candida albicans* ATCC 10231, and *Candida albicans* 6853 (clinical isolate). All strains were grown on a nutritious agar medium and incubated for 18–20 h at 37 °C in order to obtain fresh cultures.

For the qualitative screening of the antimicrobial efficiency, the spot diffusion method was used according to the CLSI adapted standard. Microbial suspensions of 1.5 × 10^8^ CFU/mL density (corresponding with the 0.5 McFarland standard) were prepared from all microbial cultures and seeded on the Müller–Hinton agar medium (for bacterial strains) and Sabouraud agar medium (for yeast strain), followed by spotting over of 50 μL of each sample listed in the Table 2. After equal diffusion of the compound in the medium, for 15 min at room temperature, the plates were incubated at 37 °C for 18 h, in 2 different conditions: blue light (470 nm) and darkness. To avoid dehydration of the environment, humid conditions were created in the incubator. In addition, the blue light conditions were ensured by a commercial light LED source represented by a UV lamp OSRAM HQE 40 with an emission spectrum in the range of 300 nm ≤ λ ≤ 420 nm and an irradiance E = (20 ± 0.5) W/m^2^ [29]. The sensibility of tested microbial strains was evaluated by measuring the diameters of the inhibition zones that appeared around the spot.

The minimum inhibitory concentration (MIC) values of the tested compounds were determined using the serial microdilution method performed in nutritive broth that was added in sterile 96-well plates. For each tested compound, including the controls, binary dilutions were performed, covering a range of concentrations between 100 µM and 0.195 µM (calculated for Curcumin). Further, 15 μL of microbial suspension, adjusted to 1.5 × 10^7^ CFU/mL, were added in each well. The MIC values were established by considering the higher compound dilution that inhibited microbial growth and multiplication. Furthermore, to confirm the MIC values, the total number of microbial cells (CFU/mL) in the well corresponding to the MIC was determined using the viable cell count (VCC) method. Each experiment was performed in triplicate and repeated on at least three separate occasions.

#### 2.2.8. Statistical Analysis

The experiments were performed in triplicate, and the data are presented as means and standard deviations ± SD. Differences between the samples were assessed by one-way ANOVA and *T*-tests using MS Excel 2010 from Microsoft (Redmond, WA, USA). Statistical significance was considered at *p* < 0.05.

## 3. Results

### 3.1. Critical Micelle Concentration

Polymeric micelles were prepared to be used as carriers for Curcumin in the light-assisted inhibition of the microorganism’s growth. Three Pluronic derivatives were investigated, with various hydrophobicities, due to different lengths of the polypropylene oxide (PPO) and polyoxyethylene oxide (PEO) moieties. The chemical structures, HLB values, and determined critical micellar concentrations are listed in Table 3. The CMC were determined using Pyrene as the fluorescence probe of the microenvironment’s polarity in the surfactant solution and were found in accordance with the data reported in the literature [30].

The used Pluronic copolymers exhibit CMCs in a broad range of values, from 10^−4^ to 10^−6^ M, due to their different aggregation tendencies, derived from the extent of the hydrophilic and hydrophobic moieties. As is expected, the highest CMC was found for Pluronic P84 (1.9 × 10^−4^ M), while the two other derivatives, with longer PPO chains, exhibit smaller values in the range of 10^−6^ M. The small CMC values for P123 and F127 recommend these compounds to be used as micellar drug delivery systems. However, we included P84 in the study due to its properties as a membrane fluidizer, in spite of its unfavorable high CMC value.

### 3.2. Polymeric Micelles Characterization

Curcumin-loaded and void polymeric micelles were prepared by dissolving the Pluronics in PBS at a concentration of 1.5% (*w*/*w*). This specific concentration was chosen in order to exceed the CMC values and to possess a minimum toxicity against normal cells, as is shown in our previous studies [31]. The toxicity of Pluronic derivatives was demonstrated to be dependent on the cell type, chemical characteristics of the polymer, and time of incubation. The hydrophobicity of the Pluronics plays a distinct role in the micelles’ internalization pattern, thus strongly influencing the cytotoxicity. The derivative F127, with the highest HLB value in the series used in the present work (HLB = 22), is efficiently internalized in the cytoplasm, while the more hydrophobic Pluronics P123 (HLB = 8) and P84 (HLB = 14) are retained in the membrane and exhibit a slower transport into the cells [32]. In general, the more hydrophobic Pluronics show higher cytotoxicity compared to the hydrophilic ones, against all types of normal cells at concentrations above 10^−4^ M [33]. Thus, the selection of the Pluronic derivatives to be used as micelles material must take into account all the effects of the hydrophobicity, i.e., the influence on the CMC value (and, consequently, the stability under dilution), the size of the micellar core, and the cytotoxicity of the carrier.

The size and size distribution of micelles, both void and with Curcumin encapsulated, are summarized in Table 4.

The main value for the size of the micelles in the F127 solution was found to be 25.08 nm, a significantly higher value (*p* < 0.05) compared to the ones recorded for Pluronic P84 and P123, since the last two block copolymers possess smaller molecular weights. As is expected for polymeric solutions at rather low concentrations, all samples show moderate polydispersity, according to the PdI values, ranging from 0.056 to 0.380. The size of polymeric micelles looks similar to other data reported in the literature [30].

The encapsulation of the drug CURC into micelles results in a slight increase in the dimensions in the case of Pluronic P84 micelles. For the compounds F127 and P123, the size of CURC-loaded micelles significantly increases (*p* < 0.05) compared to void micelles, which suggests drug’s encapsulation mainly into the hydrophobic core of the micellar aggregates.

**Figure 1:**
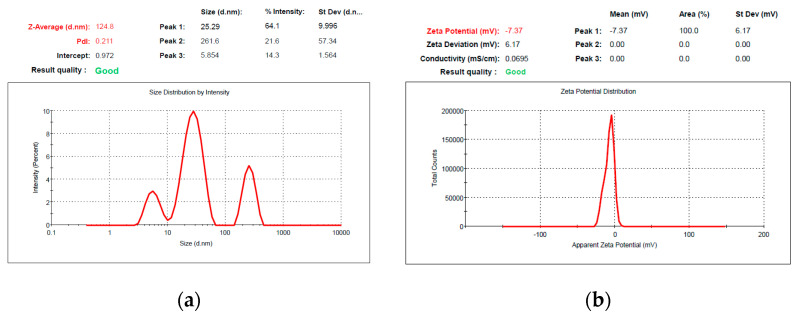
Size distribution (**a**) and zeta potential (**b**) for Pluronic P123 micellar dispersion loaded with Curcumin.

The values of zeta potential found for void Pluronic micelles are similar to those reported in the literature— −14.53 ± 0.32 mV for P84, −7.92 ± 1.51 mV for P123, and −13.93 ± 1.07 mV for F127, respectively—and no statistically significant changes were recorded after the encapsulation of the drug. Zeta potential values of the micelles with Curcumin encapsulated vary from −13.83 ± 0.32 mV for CURC-loaded P84 micelles to −7.07 ± 1.23 mV for CURC-loaded P123 micelles and to −12.40 ± 1.53 mV for CURC-loaded F127 micelles. The values are relatively low and explain the tendency of aggregation observed at high concentrations of polymer in solution. In Figure 1, the DLS diagrams for size distribution and zeta potential measured for P123 micellar dispersion with CURC encapsulated are shown as representative examples.

The DLS diagram exhibits a major signal at 25.29 nm, which corresponds to the micelles with encapsulated Curcumin, while the signal at 5.8 nm corresponds to the unassociated polymeric chains. The signal located at the higher value of 261 nm is probably due to the aggregation of micelles, which is reported in other papers, in particular for Pluronic derivatives with low zeta potential values. For the two others derivatives P84 and F127, similar behavior was recorded.

**Figure 2:**
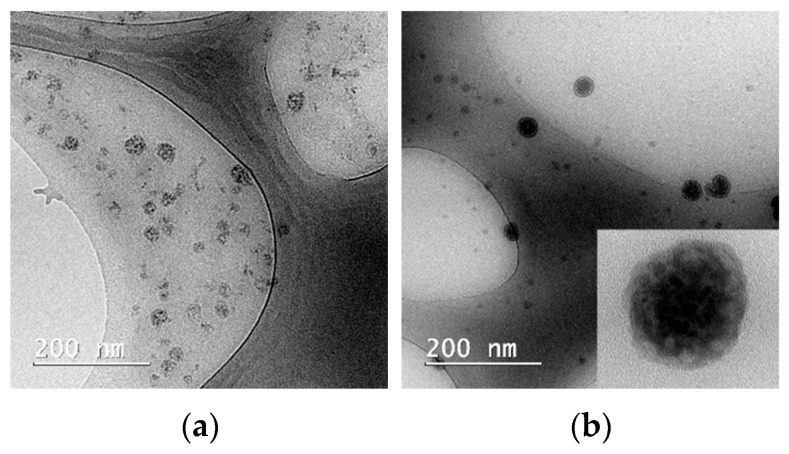
Representative TEM images of samples of void (**a**) and CURC-loaded P123 micelles (**b**).

In samples with all three Pluronic derivatives, the micelles are spherical in shape, as shown in Figure 2. The solubilization of the Curcumin in micelles does not change the shape of the aggregates in the case of all three polymers P84, P123, and F127, and the TEM images of samples with drug loaded carriers does not reveal any other polymeric nanostructures such as worm-like micelles or vesicles.

A selected concentration of polymers in solutions of 1.5% was chosen to exhibit the minimum toxicity against normal mammalian cells, but at the same time, to exceed the CMC value in order to allow for the existence of micelles. Another advantage of this low concentration is that all three Pluronic derivatives produce small spherical micelles, none of them showing transitions to worm-like micelles or vesicles.

**Figure 3:**
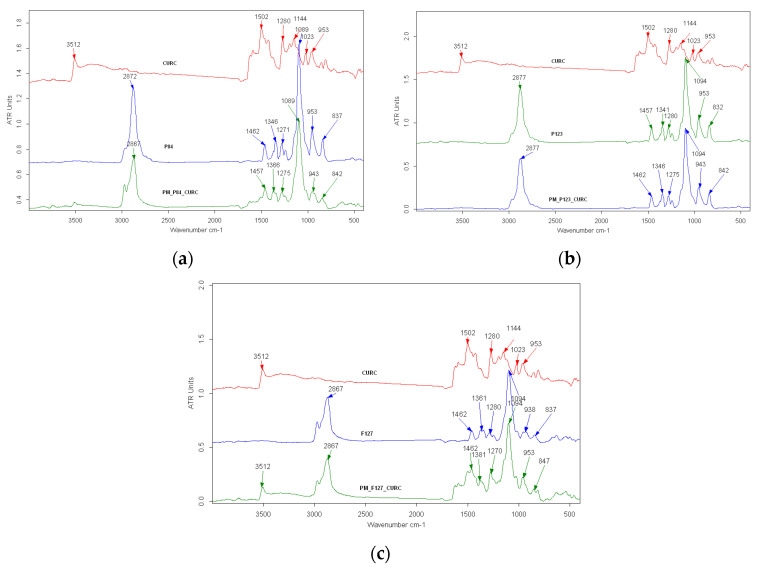
FTIR spectra of polymeric micelles with and without encapsulated Curcumin (**a**) Pluronic P84; (**b**) Pluronic P123; (**c**) Pluronic F127.

The possible interactions between drug molecules and polymer in the CURC-loaded Pluronic micelles were investigated by using FTIR spectroscopy, and the spectra are shown in Figure 3a–c.

In the FTIR spectra of pure Curcumin (Figure 3), a sharp peak at 3512 cm^−1^ is present, which corresponds to the stretching vibration of the phenolic –OH group. The peak at 1625 cm^−1^ can be associated with the –C=C– and –C=O vibrations, and the band at 1593 cm^−1^ with the symmetric aromatic ring stretching vibrations –C=C–. There are also present peaks at 1502 cm^−1^, corresponding to the –C=O vibration, and at 1023 cm^−1^, corresponding to the C–O–C stretching vibration.

Due to similar chemical compositions, FTIR spectra of freeze-dried Pluronic micelles contain the same specific peaks. The intense band located at 1089 cm^−1^ is attributed to the C–O–C vibration, while the peaks at 2867 and 2978 cm^−1^ are related to the asymmetric and symmetric stretching vibrations of C–H.

All three spectra of Pluronic micelles loaded with CURC look similar, containing all specific peaks of Pluronic derivatives and preserving most of the bands characteristic for CURC, without significant shifting. Thus, the presence of the bands associated with the main functional groups of Curcumin confirms the encapsulation of the drug into micelles, without changes in chemical bonds. The attenuation in the intensity of the peaks at 3512 cm^−1^ suggests the formation of the intermolecular hydrogen bonds between the Curcumin –OH groups and POE groups in Pluronic corona, as is reported for the behavior of CURC in other polymeric matrices containing polyoxyethylene groups [34].

### 3.3. Drug Loading and Entrapment Efficiency

One of the main drawbacks of Curcumin is its low solubility in water (less than 20 µg/mL); thus, the solubilization in micellar dispersions proves to be a viable solution to increase the drug’s solubility in aqueous formulations. A significant increase in CURC’s solubility, up to 3 mg/mL was reported in the literature in Pluronics P105, P108, and F127 solutions at concentrations above 10^−2^ M [35]. Unfortunately, these concentrations are usually cytotoxic for most normal animal cells.

The presence of the block copolymer micelles leads to increased solubility of the Curcumin in aqueous solutions, as is observed from the enhancement of the fluorescence emissions of the CURC (Figure S1).

The encapsulation of CURC in a Pluronic micellar solution at low concentrations (1% *w*/*v*) was evaluated at room temperature, and the encapsulation efficiency (EE%) was found to range from 31.2 ± 3.52 for Pluronic P123 and 43.7 ± 3.29 for Pluronic F127. The encapsulation capacity is significantly lower for P123, while the derivatives with higher HLB values, P84 and F127, show similar values of EE%: 42.7 ± 1.69 for P84 and 43.7 ± 3.29 for F127. The values for drug-loading efficiency (DL%) at the drug–polymer ratio 1:20 (*w*/*w*) were found to be 4.39 ± 0.53% for P84, very close to the value for F127 4.48 ± 0.46%, while for P123, a value of 3.71 ± 0.57% was obtained.

The drug-loading and entrapment efficiency are influenced by several factors, such as polymer concentration, the length of the hydrophobic core-forming block in the macromolecule, and the extent of the PEO corona. Various mechanisms have been proposed for the encapsulation of hydrophobic drugs, such as Curcumin, into micelles, in particular chemical conjugation or physical entrapment. The physical entrapment of drug molecules in micellar aggregates is due to the existence of intermolecular interactions: hydrophobic, van der Waals interactions, or hydrogen bonds. Curcumin’s chemical structure suggests that the formation of hydrogen bonds between the oxygen in the ether groups of PEO chains and hydroxyl groups in CURC play an important role in the drug’s encapsulation in Pluronic micelles, beyond the van der Waals interaction in the PPO core of the micelles. According to the dimension of the PPO block in the Pluronic molecule, P84 is expected to encapsulate less Curcumin than the P123 and F127 derivatives. The same PPO length in P123 and F127 suggests that the two micellar systems exhibit a similar solubilization capacity. The higher encapsulation efficiency of Pluronic F127 is due to the longer length of the PEO block, which generates a larger oxyethylene corona.

### 3.4. In Vitro CURC Release

**Figure 4:**
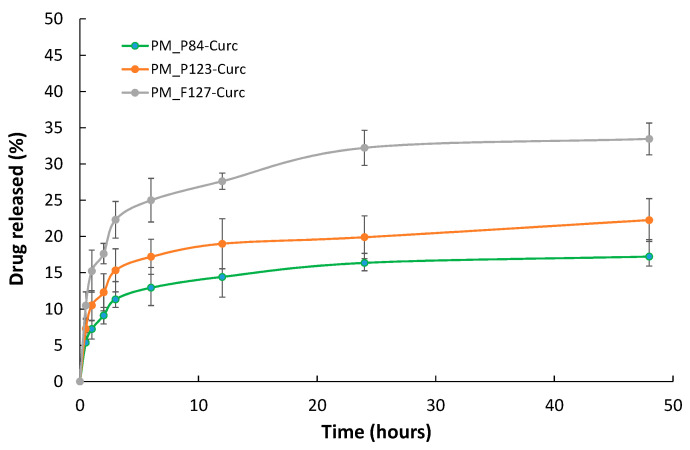
The CURC release profile from micellar solutions of various Pluronic derivatives. Data expressed as the mean value and standard deviation of three experiments.

The drug release from polymeric micelles was evaluated using the dialysis method, and the results are presented in Figure 4.

The cumulative released amount of CURC at 48 h was found to be 17.23% from micelles of Pluronic P84, while from P123 micelles, it was 22.27%, and from F127 micelles, 33.46%. Almost half of the total released drug left the micelles within the first hour, and a plateau of CURC released appears at 12–24 h. Most of the initially encapsulated CURC remain entrapped in the micelles for up to 48 h for all three Pluronic derivatives. Thus, one could consider that the Pluronic micelles exhibit a sustained release of Curcumin content.

### 3.5. Photophysical Properties of Curcumin Encapsulated in Pluronic Micelles

#### 3.5.1. Absorption and Fluorescence Spectra

**Figure 5:**
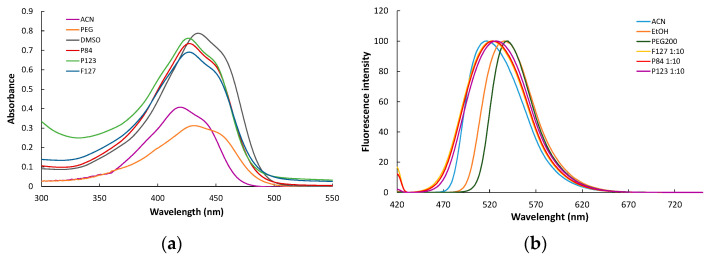
UV-VIS (**a**) and fluorescence (**b**) spectra of CURC encapsulated in various micellar systems and dissolved in various solvents.

For hundreds of years, Curcumin was extensively used as a natural dye, based on its specific optical properties, while only in the last decade has there been an interest in the fruition of these properties in other fields, such as photosensitizerz in PDT. The aqueous solution of Curcumin shows a broad absorption peak around 430 nm, related to the lowest (π−π*) transitions of conjugated CURC, and a shoulder at 355 nm, produced by the transitions (π−π*) in the feruloyl groups, which is considered as a signature of the interaction of CURC molecules with water environments [36]. In solvents with lower polarity, such as dimethyl sulfoxide (DMSO) and acetonitrile (ACN), the CURC spectra exhibit a sharper peak, with a maximum absorption at 435 nm and 420 nm, respectively (Figure 5a). When encapsulated in polymeric micelles, Curcumin shows a maximum absorption at 428 nm for all three Pluronic derivatives, which indicates the solubilization in a more hydrophobic media, i.e., the core region of micelles. This alteration of the spectra of Curcumin in other surfactant solutions was reported for ionic or nonionic amphiphiles Tween 80, Triton X100, and CTABr [37].

The fluorescence spectra of Curcumin in various solvents, heterogeneous systems, and biomimetic systems were investigated, and the reported spectral characteristics were found to be significantly influenced by the nature of the solvent, in particular, the polarity of the microenvironment. Thus, Curcumin was proposed as fluorophore in the determination of CMC values of surfactants.

In Figure 5b, representative fluorescence spectra are presented for CURC dissolved in solvents with various polarity indexes and CURC encapsulated in Pluronic micelles.

In polar solvents, such as ethanol and polyethylene glycol (PEG), the emission spectra show a maximum at 538 and 540 nm, respectively, close to the value obtained in an aqueous solution. In solvents with lower polarity, a blue shift in the emission maxima is recorded; for example, in acetonitrile, the peak is centered at 515 nm. In the polymeric micellar solutions, CURC exhibits a maximum fluorescence emission at 523 nm for micelles of P84 and F127, and a slightly red shifted maxima at 527 nm for P123, which is the most hydrophobic Pluronic used in the study. The changes in the fluorescence emission recorded for Curcumin in Pluronic micellar solutions confirm the successful encapsulation of the drug in block copolymeric aggregates. The high intensity of the fluorescence emissions of CURC in polymeric micelles compared to an aqueous solution indicates that the drug encapsulated in micelle dispersions could be used as a photosensitizer in photodynamic applications.

#### 3.5.2. Stability of Encapsulated CURC under Visible Irradiation

Curcumin was found to be a sensitive molecule, facing rapid and extensive degradation in polar media, such as in an alcoholic solution, under high temperatures and exposed to light, in particular at the radiation in the UV region. The encapsulation in more hydrophobic media, for example, in microemulsions or solid lipid nanoparticles, leads to a decrease in the degradation rate and extent. The encapsulation of CURC in micelles has proved to be an alternative to protect the drug molecule against degradation produced by irradiation. The photophysical properties of Curcumin recommend it to be used as a photosensitizer in PDT and photoinduced microbial inactivation, using various sources of radiation. Most convenient for Curcumin was considered the use of activation with light of 425, 440, or 470 nm, the last one having also been selected for the present study.

**Figure 6:**
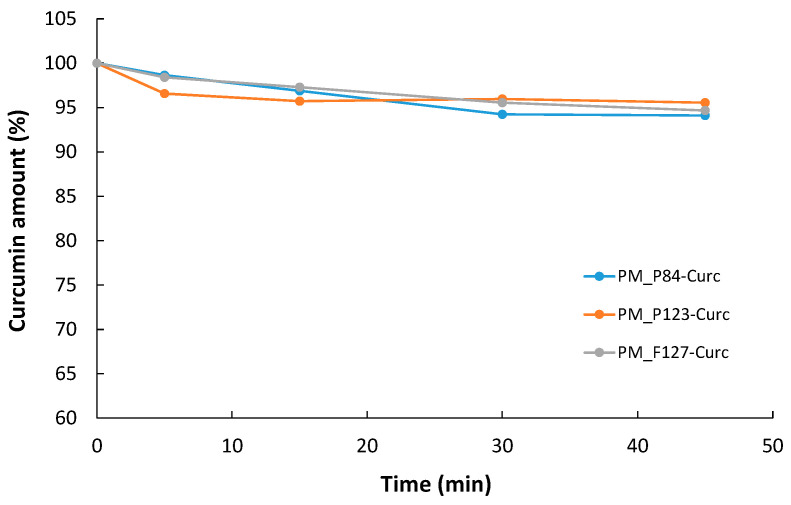
Degradation of CURC encapsulated in Pluronic micelles under visible irradiation (470 nm).

The degradation of CURC in drug-loaded micelles of different Pluronic derivatives is presented in Figure 6 as the percent of non-degraded Curcumin from the initial amount in the polymeric solution.

All three Pluronic micellar systems offer good protection against degradation, since after 45 min of exposure to the irradiation at 470 nm, only 4.4% of the initial quantity of Curcumin was degraded in P123 micelles, while in P84 and F127 micellar solutions, the degraded CURC was 5.99% and 5.32%, respectively. Thus, we presume that CURC encapsulated in block copolymeric micelles could be used in the short-term irradiation experiments without any risk of degradation and loss of the photophysical properties.

### 3.6. Influence of Curcumin-Loaded Pluronic Micelles on Microbial Membrane Permeability

#### 3.6.1. Microbial Membrane Permeability

There are many events involved in the complex interaction of Pluronic compounds with multi-drug resistant (MDR) tumoral cells, such as modification in the microviscosity of the cell membranes, due to the incorporation or attachment of the polymer molecules, reduction of the adenosine-5′-triphosphate (ATP) level, inhibition of the multidrug resistance proteins, release of cytochrome C level in cytoplasm, enhancement of the pro-apoptotic signaling, or inhibition of the drug efflux transporter, in particular P–Glycoprotein (Pgp). The interesting aspect is that these effects are most evident at the concentrations below the CMC value of the block copolymer and are diminished or absent at concentrations above CMC [38]. It was suggested that the unimers, not micelles, are responsible for the changes in tumoral cell behavior, due to their ability to penetrate the cellular membrane according to the hydrophobicity of the macromolecule. Considering the same type of mechanism of action, it is presumable that block copolymers could also have an effect in reversing antibiotic resistance in the case of microorganisms. However, very limited studies were performed on the possible influence of Pluronics on the bacterial membranes’ integrity.

To evaluate the effect of the presence of Pluronics on the membranes of bacterial and fungal cells, the accumulation of propidium iodide (PI) and 1-N-phenylnaphthylamine (NPN) as hydrophobic probes in *E. coli*, *S. aureus*, and *C. albicans* strains was measured, using both reference strains and resistant clinical isolates.

Propidium iodide is a fluorescent dye with affinity to DNA which is commonly used to evidence or quantify the dead cells, since it does not trespass the cell membrane and exhibits no accumulation in viable cells. Based on this behavior, it is also used as a marker of cellular membrane damage in both mammalian cells and microbial cultures. To evidence the alteration of the outer membrane of Gram-negative bacteria, 1-N-phenylnaphthylamine (NPN) was proposed as a fluorescent probe, with the intensity of the emitted fluorescence strongly dependent on the polarity of the microenvironment. When some chemicals affect the permeability of the outer membrane, NPN dye enters into the hydrophobic zone of the membrane, and thus, the intensity of the emitted fluorescence increases spectacularly.

**Figure 7:**
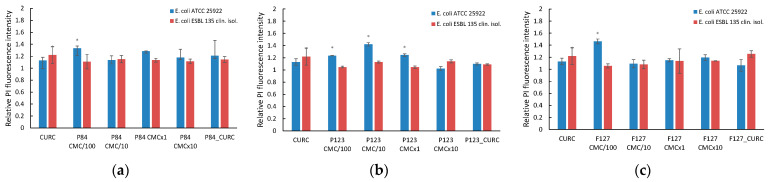
Relative PI fluorescence in *E. coli* cultures incubated with micellar and unassociated molecules of Pluronic derivatives: Pluronic P84 (**a**); Pluronic P123 (**b**); Pluronic F127 (**c**). The concentration of Curcumin solubilized in DMSO and in polymeric micellar dispersion is 100 µM. Concentrations of Pluronic derivatives were selected according to their CMC values (Table 1) to cover the micellar domain (concentrations equal to CMC and 10-fold higher) and unimer domain (concentrations equal to CMC/100 and CMC/10).

In Figure 7a–c, the variation in the relative fluorescence of PI in *E. coli* strains (*Escherichia coli* ATCC 25922 and *Escherichia coli* ESBL 135, clinical isolate) in the presence of various Pluronic derivatives is presented.

In the case of standard *E. coli* cultures treated with Pluronic solutions, an increase in membrane permeability was recorded only at very low concentrations for all types of polymer used in the study. For hydrophilic Pluronics P84 and F127, a slight increase (*p* < 0.05) in the fluorescence relative to the control samples, considered as unity, up to 1.34 and 1.46 was recorded only at concentrations 100 times lower than CMC (denoted CMC/100), when the polymeric solution contains monomer and no micelles. At higher concentrations, for both micellar and pre-micellar, no significant changes in the fluorescence of PI is recorded. A different behavior is shown by the more hydrophobic derivative P123, when the increase in the membrane permeability is observed in an extent domain of concentrations, from very diluted (100 smaller than CMC) up to micellar concentrations (CMC). The further increase in the polymer concentration up to 10 times CMC (denoted CMC×10) results in the decrease in fluorescence at values close to untreated cells. The maximum permeation of PI in the bacterial cells seems to be located at the polymer concentration one order of magnitude lower than the CMC. Similar results were reported by Batrakova et al. [39] for the hydrophobic Pluronics in the internalization of Rhodamine 123 as a membrane permeabilization marker, affecting normal and MDR tumoral cells. In the case of resistant *E. coli* cultures, no changes in cell membrane permeability was recorded over the entire domain of concentrations investigated with all three Pluronic derivatives.

**Figure 8:**
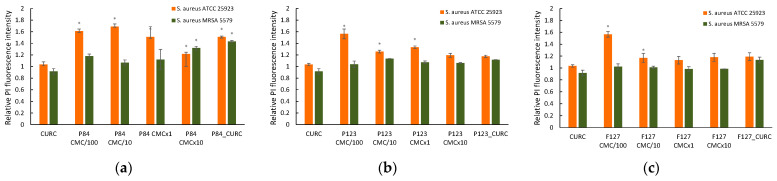
Relative PI fluorescence in *S. aureus* cultures incubated with micellar and unassociated molecules of Pluronic derivatives: Pluronic P84 (**a**); Pluronic P123 (**b**); Pluronic F127 (**c**). The concentration of Curcumin solubilized in DMSO and in polymeric micellar dispersion is 100 µM. Concentrations of Pluronic derivatives were selected according to their CMC values (Table 1) to cover the micellar domain (concentrations equal to CMC and 10-fold higher) and unimer domain (concetrations equal to CMC/100 and CMC/10).

The Gram-positive bacteria *S. aureus* proves to be more susceptible to the effect of the Pluronics on the cell membrane (Figure 8a–c). The polymer–membrane interaction in Gram-positive microorganisms is governed by the ability of the PPO block of the polymeric chain to attach to or insert into the hydrophobic phospholipidic zone of the cell wall after crossing the peptidoglycan layer of the cell wall. The situation is more complex in the case of Gram-negative bacteria, which possess also an outer membrane, composed mainly from proteins, phospholipids, and lipopolysaccharides (LPSs); thus, the binding of Pluronic molecules involves others mechanisms.

As it is expected, hydrophilic block copolymer F127 show no effect at higher concentrations, with only a small increase in the relative fluorescence measured at very low concentrations (100 time smaller than CMC). Polymer F127 is considered a non-Pgp-inhibiting member of the Pluronic family, and its role in drug delivery is restricted to the carrier-forming ability. For the standard *S. aureus* strain, an increase in the relative fluorescence emission of PI was observed when the cultures were incubated with both unassociated molecules and micellar solutions, for Pluronic derivatives P84 and P123. The exception is Pluronic F127, which shows a slight increase in PI fluorescence in the low concentration region up to CMC. The derivative with moderate HLB, hydrophilic P84, also shows the highest effect on membrane permeability at concentrations 10 times higher than CMC, and after treatment with Curcumin-loaded micelles, too.

The most intense effect is recorded, for all block copolymers, in very diluted polymeric solutions, 100 and 10 smaller than the CMC of the respective Pluronics.

The MRSA strain shows no membrane changes when exposed to a Pluronic solution over the entire concentration range. Unexpectedly, a very small increase in the PI probe’s fluorescence was recorded in the samples incubated with micelles of P84 and Curcumin encapsulated in micelles of P84, regardless of the lack of the changes recorded in the presence of Curcumin dissolved in DMSO.

**Figure 9:**
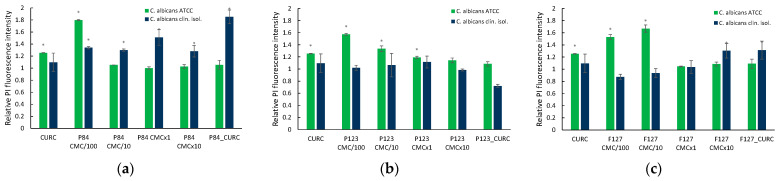
Relative PI fluorescence in *C. albicans* cultures incubated with micellar and unassociated molecules of Pluronic derivatives: Pluronic P84 (**a**); Pluronic P123 (**b**); Pluronic F127 (**c**). The concentration of Curcumin solubilized in DMSO and in polymeric micellar dispersion is 100 µM. Concentrations of Pluronic derivatives were selected according to their CMC values (Table 1) to cover the micellar domain (concentrations equal to CMC and 10-fold higher) and unimer domain (concetrations equal to CMC/100 and CMC/10).

The results obtained with *C. albicans* cultures show that the fungus exhibits higher sensitivity to the presence of the Pluronic polymers, and the membrane is more affected, compared to bacterial strains, as is demonstrated by the changes in the PI fluorescence (Figure 9a–c).

In the case of *Candida albicans* ATCC 10231 incubated with all three Pluronics, membrane permeability was increased. For the hydrophilic derivatives P84 and F127, the increase in the relative fluorescence intensity is in the range of 1.2–1.8, at the lowest concentration of polymer in solution (100 lower than CMC values). The highest effect is observed for the polymer P84, with an HLB value of 14 and the smallest molecule among the tested block copolymer, at a concentration 100 times smaller than the CMC value. A significant increase in membrane permeability is also observed for Pluronic F127 at low concentrations (100 times and 10 times smaller than the CMC value of the polymer), as demonstrated by the values recorded for relative fluorescence intensity (1.52 and 1.66, respectively).

The more hydrophobic Pluronic P123 shows efficiency in membrane damaging on the largest domain of concentration, up to CMC. The highest increase in PI fluorescence is recorded at the lowest concentration (100 times smaller than CMC) when only monomeric species are present.

The resistant *C. albicans* strain is less influenced by the incubation with Pluronic solutions, for the polymers P123 and F127, with high molecular weights. As an exception, a slight effect is observed in the case of cultures treated with concentrated F127 solution (10 times higher than CMC) and the related micellar solution loaded with Curcumin. The Pluronic P84 with hydrophilic characteristics, but a smaller molecule than P123 and F127, proved to be effective over the whole domain of concentrations. An unexpected high effect was recorded for the micellar solution with Curcumin encapsulated, despite the same concentration of CURC dissolved in DMSO showing no change to the fluorescence of PI.

It is to be noted that also in the case of *Candida albicans* ATCC 10231, the Curcumin seems to be effective, producing a slight increase in membrane permeability (1.26 relative fluorescence intensity), while no such effect could be observed in the case of clinical isolate *Candida albicans* 6853.

#### 3.6.2. Outer Membrane Permeabilization

The differences in the behavior of Gram-negative and Gram-positive bacteria in the presence of various antimicrobial agents are due to the presence of the outer membrane, consisting mainly of lipopolysaccharide (LPS) in the first case. This organelle acts as a barrier that protects Gram-negative bacteria against the aggression of antibiotics existing in the extracellular environment. For a certain chemical compound to actively interact with the bacterial membrane, it is mandatory to first trespass the outer layer of LPS. The hydrophobic NPN dye exhibits an increase in fluorescence when it is located in the phospholipid bilayer. Thus, the NPN uptake is considered a direct measurement of the outer membrane’s integrity, since an intact one prevents the penetration of the compound into the hydrophobic area of the bacterial membrane.

**Figure 10:**
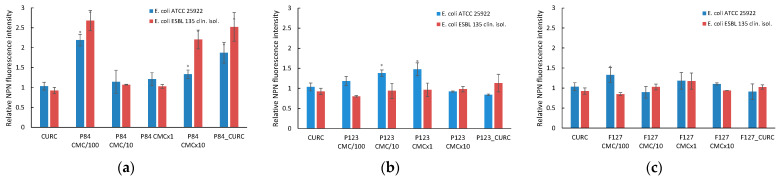
Relative NPN fluorescence in *E. coli* cultures incubated with micellar and unassociated molecules of Pluronic derivatives: Pluronic P84 (**a**); Pluronic P123 (**b**); Pluronic F127 (**c**). The concentration of Curcumin solubilized in DMSO and in polymeric micellar dispersion is 100 µM. Concentrations of Pluronic derivatives were selected according to their CMC values (Table 1) to cover the micellar domain (concentrations equal to CMC and 10-fold higher) and unimer domain (concentrations equal to CMC/100 and CMC/10).

The strain of reference and resistant *E. coli* were incubated with the Pluronic derivatives, and the results of the NPN fluorescence modification is presented in Figure 10.

The hydrophilic Pluronic F127 with high molecules induces modification of the outer membrane of the reference *E. coli* ATCC 25922 only at the lowest concentration (CMC/100), while the Pluronic P84, with smaller molecules, produces the permeabilization of the outer membrane over the whole domain of concentrations, in the presence of both micelles and unassociated molecules.

For the hydrophobic derivative P123, the effect is evidenced only at concentrations near the CMC values.

In the experiments with *Escherichia coli* ESBL, NPN uptake was increased only when incubated with Pluronic P84, while no changes in the fluorescence intensity were recorded for samples with P123 and F127. Very peculiar behavior was registered with the Pluronic P84 solution, with the highest increase in the relative fluorescence of NPN (more than twice compared to the untreated control bacterial cultures) for the smallest concentration of polymer (CMC/100) and the highest one (10 times highest than CMC) and the corresponding micellar solution loaded with Curcumin.

The ability of CURC to induce cell membrane modifications was investigated for various microorganisms. Unfortunately, the effect on the integrity and fluidization of the bacterial membrane was investigated with various methods (TEM and SEM images, flow cytometry, confocal microscopy, spectrofluorimetry), and experimental conditions vary in a large domain; thus, the reported results are contradictory. For example, an increase of 47% for PI fluorescence intensity in *E. coli* cells treated with high concentrations of CURC was determined by using flow cytometry [40].

Furthermore, the CURC effect on membrane fluidization was found to be time dependent, and an increase of the fluorescence of 54% was measured using a concentration of 500 µg/mL (7-fold higher than the concentration used in our study), after 24 h of incubation [41]. Oppositely, in another study performed with CURC solutions in DMSO at various concentrations 0.25–2.5 mM, with or without irradiation at 470 nm, the authors concluded that CURC does not produce membrane permeabilization [42]. A higher increase in PI fluorescence intensity produced by 100 µg/mL of CURC was produced in *S. aureus* cultures, incubated for 2 h, while the fluorescence in *E. coli* cells was found to increase after 30 min of incubation, but disappear after 2 h [43].

In conclusion, in the experimental conditions of our study, at low concentrations and short incubation times, CURC in DMSO did not influence the permeability of the microbial cell membranes in *C. albicans* clinical isolates and both types of *S. aureus* strains. A moderate effect was recorded in the fluidization of the membrane for standard *C. albicans* and for both standard and resistant *E. coli.* However, the effect of CURC on the cellular membrane was lost when encapsulated in Pluronic micelles.

Regarding the effect of the block copolymer solutions on the microviscosity of the microbial cell membrane, there are very few papers reporting the variation in the fluorescence intensity of standardized probes PI and NPN. The effect of the micelles on membrane integrity was absent in the majority of published papers, considering that only the monomeric species of surfactant produce the membrane fluidization. Experiments performed with highly concentrated Pluronic F127 on *P. aeruginosa* and *E. coli* suggested that polymeric micelles do not induce membrane permeability, based on tetraphenylethylene fluorescence, determined by using confocal microscopy. However, Bondar et al. [44], in a study on the effect of novel glycerol-based trifunctional block copolymers (TBC) of propylene oxide and ethylene oxide and Pluronic L61 on the cell’s plasma membrane, suggest that these compounds are also able to produce membrane damage at increased concentrations above CMC, since the micellar aggregate may fuse with the membrane and disrupt it. A possible explanation for the results that we obtained in some microbial strains with micellar solutions is due to the ability of Pluronic aggregates to encapsulate the hydrophobic dye and increase cellular uptake, without directly damaging the membrane. Such mechanisms for penetration in the bacteria, with a first step of the attachment of the carrier to the bacterial wall, followed in a second step by the disruption of the peptidoglycan layer and incorporation of the particle inside the microorganism, were proposed for many drug delivery systems as an explanation for the increased cellular uptake. The interaction of Pluronics with cellular membranes is very complex, being different in the case of monomeric species and micellar aggregates. In addition, the complexity of the interaction must be taken into account in view of the fact that block copolymers interact with the microdomains of cell membranes in a different way, hence the very large variation in membrane fluidization effects at different concentrations, also depending on the membrane composition in various types of cells.

### 3.7. Photoinduced Antimicrobial Activity of Curcumin-Loaded Pluronic Micelles

The results regarding the qualitative screening of the antimicrobial activity of the tested samples are shown in Table 5 (and Figures S2–S4 in Supplementary materials) as diameters of the inhibition zone.

As a general consideration, the CURC sample dissolved in DMSO (Curcumin control) expressed an inhibition zone toward all tested microbial strains in both conditions (darkness and 470 nm light), with higher diameters for *C. albicans* strains in the 470 nm light incubation condition. The lower efficiency was recorded in the growth control of *E. coli* in both standard and resistant strains, incubated in dark or irradiated. For the samples PM P84_CURC, enhanced antimicrobial activity can be highlighted when the incubation was performed in the presence of Gram-positive strains *S. aureus*, while a moderate effect was found against *E. coli* and *C. albicans* strains. For CURC encapsulated in the other two polymeric micelles P123_CURC and PM F127_CURC, the inhibition zones are in the range 8–10 nm, with a minor increase for the effect of P123_CURC on resistant E. coli and for PM F127_CURC on resistant C. albicans, under irradiation.

Instead, the results reveals that samples containing similar CURC amounts in unassociated molecule Pluronic solutions (concentrations below CMCs), namely, Premicellar P84_CURC, Premicellar P123_CURC, and Premicellar F127_CURC, expressed large inhibition zones toward *S. aureus* Gram-positive strains and *E. coli* strains, while significantly higher values are obtained for *C. albicans* strains when the incubation was performed in 470 nm light, compared with the darkness condition.

All the tested samples that contain Pluronic compound (considered as control samples) expressed no inhibition zone after the contact with the tested microbial strains in the same two incubation conditions (Table S1 in Supplementary materials).

In order to demonstrate the light activation of Curcumin and to quantify the antimicrobial activity of the tested samples, following the incubation in the same two conditions (470 nm light and darkness), the MIC values have been determined and included in Table 6.

None of the tested Pluronic derivatives (Pluronic^®^ P84, Pluronic^®^ P123, and Pluronic^®^ F127) exhibited antibacterial activity against all microbial strains over the concentration domain used in the study (Table S2 in Supplementary materials).

As it is shown in Table 6, the quantitative results confirm the qualitative results regarding the efficiency of the samples Premicellar P84_CURC, Premicellar P123_CURC, and Premicellar F127_CURC after 470 nm light incubation, the MIC values being lower compared to those obtained in darkness incubation conditions for the same microbial strains *S.aureus* and *C. albicans*.

In addition, the increase in the efficiency of the samples Premicellar P84_CURC, Premicellar P123_CURC, and Premicellar F127_CURC against *Candida* strains can be observed after incubation in 470 nm light, for which MIC values of 6.25 µM–2.5 µM were obtained, compared to the control sample of Curcumin, for which the MIC values were 25 µM.

The MIC values obtained in the case of incubation with premicellar Pluronic solutions containing CURC confirm the qualitative results of inhibition zones. Some differences were observed in the behavior of CURC-loaded polymeric micelles. In this case, the two experiments could not be compared since the MIC values could only be estimated to be higher than 100 µM, because this is the maximum concentration of CURC possible to be encapsulated in polymeric micelles. This could explain, for example, the apparent contradiction in the behavior of CURC-loaded P84 micelles, which shows a moderate radius of the inhibition zone, with various values according to the incubation conditions, while no difference between the MIC values could be observed. Additionally, the differences between the antibacterial activity reported as an inhibition zone and the minimum inhibitory concentration are due mainly to the different testing protocols for quantitative and qualitative antimicrobial measurements. In the first experiment, CURC-loaded carriers ensure contact with the microorganism grown at the surface of the solid medium, while in the second experiment, the CURC-loaded carriers interact with the microorganism in the volume of the liquid medium. Thus, it is presumable that CURC encapsulated in polymeric micelles exhibits different behavior in these two experimental conditions, due to the 2D and 3D diffusion (on the surface and in the volume of the nutrient solution). The differences were attenuated in the experiments involving free CURC in Pluronic solutions containing unassociated molecules.

**Figure 11:**
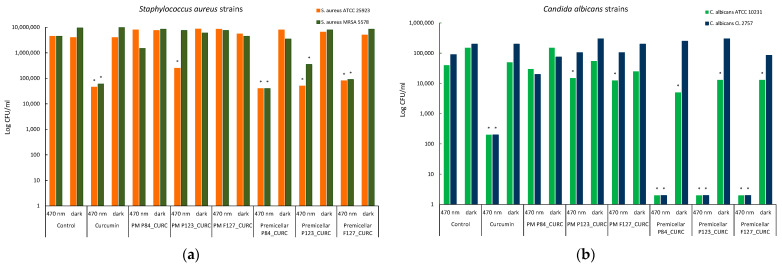

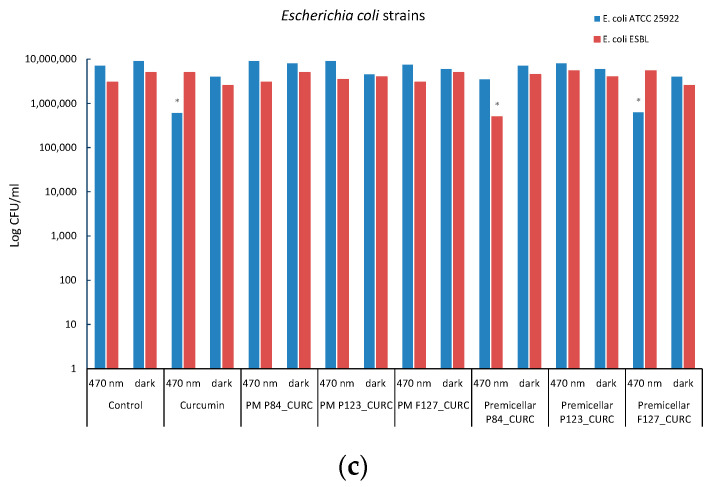
Graphical representation of the log10 values of colony forming units (CFU)/mL representing the viable cells of *S. aureus* (**a**), *C. albicans* (**b**), and *E. coli* (**c**) strains, after incubation in two different conditions: blue light (470 nm) and darkness.

All these results have been confirmed by the total number of microbial cell value (CFU/mL) determinates in the well corresponding to the MIC, using the viable cell count (VCC) method. The logarithmic representation of these values shows significant differences between the results obtained for Premicellar P84_CURC, Premicellar P123_CURC, and Premicellar F127_CURC samples in the two incubation conditions, with differences of at least two logarithmic units in the case of the same two microbial species: *S aureus* strains (Figure 11a) and *C. albicans* strains (Figure 11b).

In the case of *C. albicans* cultures, the photoinactivation effect produced by CURC is also correlated with the results of bacterial cell membrane permeabilization in the presence of Pluronic derivatives, especially in the premicellar area.

The lack of inhibitory activity against Gram-negative strains of *E. coli*, in the condition of this experiment, was also observed after the MIC determination test and confirmed by the viable cell count method (VCC method) (Figure 11c). Even if the block copolymer P84 produces some increase in membrane permeability in the case of *E. coli*, this is not sufficient to ensure the photoinhibition effect under the conditions of using a low concentration of Cucumin.

Literature surveys show a large variety of MIC values reported for free Curcumin, usually dissolved in DMSO, against common pathogens. For example, for *E. coli*, values ranging from 8 to 156 µg/mL were found, probably due to the MIC dependence of the experimental conditions. In the conditions selected for our study, we obtained for a reference sample CURC dissolved in DMSO against standard bacterial cultures, 50–100 µg/mL for *S. aureus* and *C. albicans*, similar MIC values to those reported in literature.

Moreover, the comparison between the efficiency proved by CURC encapsulated in various drug delivery systems based on published data becomes more difficult [45].

In some cases, the encapsulation of the antibiotic into micellar carriers from Pluronic enhances the antibacterial efficiency, but in other experiments, it does not produce any change in the biological effect [46].

In the present work, samples with CURC encapsulated in polymeric micelles exhibit a reduction in the antibacterial effect compared to the efficiency of the same concentration of CURC dissolved in DMSO, for all three Pluronic derivatives. The reduction in the photoinactivation efficiency is probably due to the lower extent of free CURC molecules able to interact with the cell membrane, since most of the drug is trapped in the micellar core.

The decrease in the photoinactivation efficiency of CURC encapsulated in surfactant micelles compared to the free CURC (dissolved in DMSO) was also reported by Ryu et al. [47]. The encapsulation of Curcumin in gemini surfactant Surfynol 465 or Tween 80 micelles leads to a reduction in photosensitizing efficiency against *Escherichia coli* O157 : H7 compared to CURC in DMSO.

Similar results have been obtained by using rather close concentrations of CURC-Pluronic micelles (100 µg/mL), showing that encapsulated Curcumin produces a lower reduction for *C. albicans* of 4.28 log compared to the one produced by free CURC in DMSO (4.97 log). Other studies report more effective photoinactivation of microorganism, but using very concentrated CURC drug carriers (500 µM) and long-term exposure (up to 24 h), which is not feasible for clinical applications [48].

A very interesting point of behavior must be noted for samples of CURC in premicellar solutions of Pluronic derivatives, where a significant increase in the growth reduction of *C. albicans* cultures, for both standard and clinical isolate strains, was determined. These results well correlate with the findings in the measurement on cell membrane permeability, while the increase in fluidization produced by micelles (Figure 9) is not reflected in an enhancement of antifungal activity.

Even if the photoinhibitory efficiency of CURC in Pluronic micelles is not superior to that presented by free CURC, encapsulation in polymeric micelles brings other benefits, such as increasing stability, bioavailability, and drug release kinetics. Further research is needed to elucidate the effects that the presence of Pluronic monomers in the carrier can bring, and the possibility of increasing the amount of encapsulated drug while maintaining the lowest possible amount of micelle-forming polymer.

## 4. Conclusions

The present work demonstrated the possibility of producing a safer, cost-effective carrier for the encapsulation of CURC using Pluronic derivatives, and proposed a protocol for the photoinactivation of microorganisms using blue light short-term irradiation.

The study also aimed to gain an insight into the influence of the physico-chemical characteristic of Pluronic derivatives on the membrane permeabilization of standard and resistant bacteria and yeasts.

Curcumin-loaded polymeric micelles have been prepared using three Pluronic derivatives with different lengths of hydrophobic and hydrophilic blocks. The polymer concentration was chosen to exceed the CMC value in order to allow that formation of the micellar aggregates, but at the same time, was sufficiently low to exhibit minimum cytotoxicity against normal cells and reduce the environmental impact. The encapsulation of drugs into the hydrophobic core of the Pluronic micelles leads to an increase in the fluorescence emission and enhances the stability when the samples were exposed to the blue light; thus, it is presumable that CURC micelles can be successfully used as a photosensitizer in microbial photoinactivation.

The in vitro study with NPN and PI as fluorescent probes showed that Pluronic derivatives P84 and P123 enhanced cellular uptake at submicellar concentrations or low concentrations near CMC values of polymers. No significant effect of membrane fluidization was found for Pluronic F127 solutions. The Pluronic micelles did not produce changes in the membrane permeability of microbial strains, except P84 in *S. aureus* and *C. albicans* standard cultures.

The evaluation of the antimicrobial activity against reference and clinical isolate strains of *E. coli*, *S. aureus*, and *C. albicans* demonstrates that CURC loaded into Pluronic micelles shows a slightly lower efficiency compared to free CURC dissolved in DMSO, in both dark and irradiation conditions. The efficiency of the CURC-loaded polymeric micelles as a photosensitizer was tested at sub-MIC concentrations using irradiation at 470 nm, considered a very safe light exposure for normal cells. In these moderate conditions, no reduction in the bacterial growth was recorded for both *E. coli* cultures. Our results show that the Gram-positive bacteria and fungi were more susceptible to the PDT treatment, with moderate results for the reduction in the bacterial growth of *S. aureus* for CURC in all block copolymer derivatives, and very good results in the case of *C. albicans* treated with submicelar solution of Pluronics.

More studies need to be performed on the optimization of drug-loading efficiency, in order to achieve a formulation with better efficiency in conditions of low polymer concentrations and short-term exposure to the irradiation, suitable for clinical applications. Additionally, further work is required to elucidate the complex interaction of micellar systems with the bacterial cell membranes, in order to better understand and valorize the influence of various types of block copolymers as membrane fluidizers.

## Figures and Tables

**Table 1:** Composition of the Pluronic and CURC solutions investigated as membrane fluidizers.

Sample Code	Curcumin Concentration (μM)	Pluronic Concentration (μM)	Solvent
P84_CCMx10	-	1900	PBS
P84_CCM	-	190	PBS
P84_CCM/10	-	19	PBS
P84_CCM/100	-	1.9	PBS
P123_CCMx10	-	88	PBS
P123_CCM	-	8.8	PBS
P123_CCM/10	-	0.88	PBS
P123_CCM/100	-	0.088	PBS
F127_CCMx10	-	79	PBS
F127_CCM	-	7.9	PBS
F127_CCM/10	-	0.79	PBS
F127_CCM/100	-	0.079	PBS
CURC_P84	100	3.57	PBS
CURC_P123	100	2.60	PBS
CURC_F127	100	1.19	PBS
CURC_DMSO	100	-	DMSO

**Table 2:** Sample composition and codification.

Sample Code	Content
DMSO	DMSO (Dimethyl sulfoxide)
CURC	100 μM Curcumin in DMSO
PM_P84	P84 1.5%
PM P84_CURC	100 μM Curcumin in P84 1.5%
PM_P123	P123 1.5%
PM P123_CURC	100 μM Curcumin in P123 1.5%
PM F127	F127 1.5%
PM F127_CURC	100 μM Curcumin in F127 1.5%
DMSO-premicellar P84	1 mL DMSO + 1 mL P84 4 × 10^−4^ M
Premicellar P84_CURC	2 mL 200 μM Curcumin in DMSO + 2 mL P84 4 × 10^−4^ M
DMSO-premicellar P84	1 mL DMSO + 1 mL P123 1.8 × 10^−5^ M
Premicellar P123_CURC	2 mL 200 μM Curcumin in DMSO + 2 mL P123 1.8 × 10^−5^ M
DMSO-premicellar P84	1 mL DMSO + 1 mL F127 1.6 × 10^−5^ M
Premicellar F127_CURC	2 mL 200 μM Curcumin in DMSO + 2 mL F127 1.6 × 10^−5^ M

**Table 3:** HLB and CMC values of Pluronic derivatives used for micelles formation.

Pluronic Derivative	Structural Formula	MW	HLB	CMC ^1^
Pluronic P84	(PEO)_19_-(PPO)_43_-(PEO)_19_	4200	14	1.9 × 10^−4^ M
Pluronic P123	(PEO)_18_-(PPO)_62_-(PEO)_18_	5750	8	8.8 × 10^−6^ M
Pluronic F127	(PEO)_95_-(PPO)_62_-(PEO)_95_	12600	22	7.9 × 10^−6^ M

^1^ The CMC values are experimentally determined.

**Table 4:** Physico-chemical characteristics of Pluronic micelles with and without encapsulated Curcumin at 37 °C (values are mean ± SD, *n* = 3).

Sample	Composition	Size (nm)	PdI
M1	Micelles Pluronic P84 in PBS	18.00 ± 0.38	0.380 ± 0.017
M2	Micelles Pluronic P123 in PBS	19.69 ± 0.43	0.056 ± 0.010
M3	Micelles Pluronic F127 in PBS	25.08 ± 1.83	0.164 ± 0.035
P1	CURC-loaded Micelles Pluronic P84 in PBS	19.27 ± 2.04	0.323 ± 0.097
P2	CURC-loaded Micelles Pluronic P123 in PBS	25.50 ± 0.57	0.156 ± 0.011
P3	CURC-loaded Micelles Pluronic F127 in PBS	28.06 ± 0.77	0.268 ± 0.063

**Table 5:** The diameter values of the inhibition zone expressed by the tested samples that contain Pluronic and curcumin compounds.

Samples Code	Incubation Conditions	Inhibition Zone Diameters (mm)					
		*S. aureus* ATCC 25923	*S. aureus* MRSA 5578	*E. coli* ATCC 25922	*E. coli* ESBL 135	*C. albicans* ATCC 10231	*C. albicans* CL 2757
DMSO control	470	0	0	0	0	0	0
	darkness	0	0	0	0	0	0
CURC control	470	10	12	9	10	15	15
	darkness	8	11	8	9	10	12
PM P84_CURC	470	13	14	10	15	12	12
	darkness	12	13	10	12	10	10
PM P123_CURC	470	10	12	10	14	10	10
	darkness	0	10	10	0	0	11
PM F127_CURC	470	10	10	10	10	8	15
	darkness	9	8	10	10	8	10
Premicellar P84_CURC	470	10	10	10	10	20	13
	darkness	9	10	10	10	10	10
Premicellar P123_CURC	470	10	10	10	10	20	15
	darkness	9	10	10	10	10	10
Premicellar F127_CURC	470	10	12	10	10	15	13
	darkness	10	10	8	8	10	10

**Table 6:** Minimal inhibitory concentration of tested Pluronic and Curcumin compounds.

Samples Code	Incubation Conditions	Minimal Inhibitory Concentration (µM)					
		*S. aureus* ATCC 25923	*S. aureus* MRSA 5578	*E. coli* ATCC 25922	*E. coli* ESBL 135	*C. albicans* ATCC 10231	*C. albicans* CL 2757
Curcumin control	470	50	50	100	100	25	25
	darkness	100	100	>100	>100	100–50	100–50
PM P84_CURC	470	100	100	>100	>100	>100	>100
	darkness	100	100	>100	>100	>100	>100
PM P123_CURC	470	100	100	>100	>100	>100	>100
	darkness	100	100	>100	>100	>100	>100
PM F127_CURC	470	75	100	>100	>100	>100	>100
	darkness	100	100	>100	>100	>100	>100
Premicellar P84_CURC	470	75	75	>100	>100	12.5	6.25
	darkness	100	100	>100	>100	>100	>100
Premicellar P123_CURC	470	75	75	>100	>100	12.5	12.5
	darkness	100	100	>100	>100	>100	>100
Premicellar F127_CURC	470	75	75	>100	>100	12.5	12.5
	darkness	100	100	>100	>100	>100	>100

## Data Availability

Not applicable.

## Supplementary Materials

The following supporting information can be downloaded at: https://www.mdpi.com/article/10.3390/pharmaceutics14102137/s1, Figure S1: Variation of the fluorescence intensity of Curcumin in polymeric micelles at different concentrations of Pluronic F127; Figure S2: Images representing the inhibition zone after blue light (left) and darkness (right) exposure of *S. aureus* strains in the presence of tested samples. A. *S. aureus* ATCC 25923; B. *S. aureus* MRSA 5579 (clinical isolate); Figure S3: Images representing the inhibition zone after 470 nm blue light (left) and darkness (right) exposure of *C. albicans* strains in the presence of tested samples. A. *C. albicans* ATCC 10231; B. *C. albicans* CL 6853 (clinical isolate); Figure S4: Images representing the inhibition zone after 470 nm blue light (left) and darkness (right) exposure of *E. coli* strains in the presence of tested samples. A. *E. coli* ATCC 25922; B. *E. coli* ESBL 135 (clinical isolate); Table S1: The diameter values of the inhibition zone expressed by the tested samples that contains Pluronic compound at micellar concetrations (1.5% *w*/*v*) and submicellar cocentrations (CMC/10); Table S2: Minimal inhibitory concentration of tested Pluronic compound at micellar concetrations (1.5% w/v) and submicellar cocentrations (CMC/10).
