# Supplementary material for: Influence of the Hydrophobicity of Pluronic Micelles Encapsulating Curcumin on the Membrane Permeability and Enhancement of Photoinduced Antibacterial Activity
Source: Pharmaceutics. 2022 Oct 8;14(10):2137. doi: 10.3390/pharmaceutics14102137 (PMC9608470; doi:10.3390/pharmaceutics14102137)
Supplement: Supplementary file 1 [file pharmaceutics-14-02137-s001.zip › pharmaceutics-1931812-supplementary.pdf]

Supplementary material

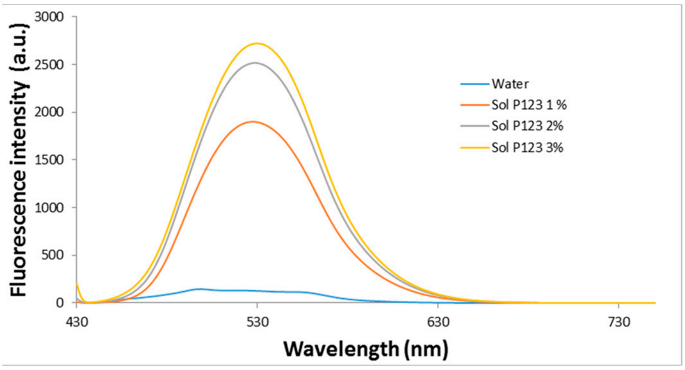

Figure S1. Variation of the fluorescence intensity of Curcumin in polymeric micelles at different concentrations of Pluronic F127. Initial concentration of Curcumin 5 μg/mL. Excitation wavelength 325 nm.

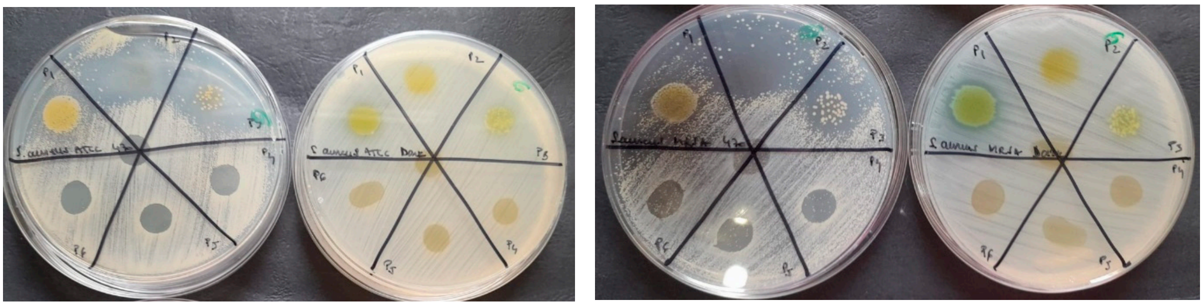

A. B.

Figure S2. Images representing the inhibition zone after blue light (left) and darkness (right) exposure of *S. aureus* strains in the presence of tested samples. (A.) *S. aureus* ATCC 25923; (B.) *S. aureus* MRSA 5579 (clinical isolate).

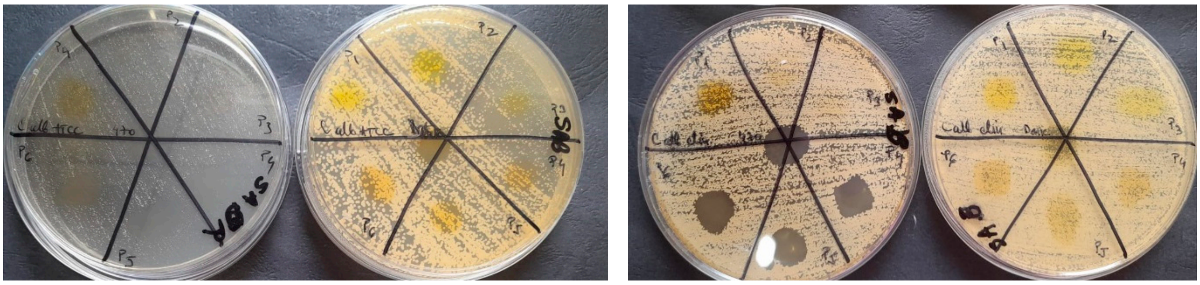

A. B.

Figure S3. Images representing the inhibition zone after 470 nm blue light (left) and darkness (right) exposure of *C. albicans* strains in the presence of tested samples. (A.) *C. albicans* ATCC 10231; (B.) *C albicans* CL 6853 (clinical isolate).

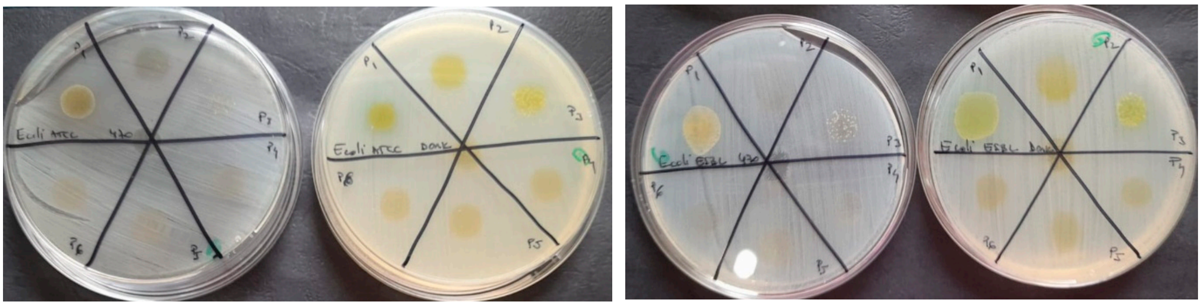

A. B.

Figure S4. Images representing the inhibition zone after 470 nm blue light (left) and darkness (right) exposure of *E. coli* strains in the presence of tested samples. (A.) *E. coli* ATCC 25922; (B.) *E. coli* ESBL 135 (clinical isolate).

Table S1. The diameter values of the inhibition zone expressed by the tested samples that contains Pluronic compound at micellar concetrations (1.5% w/v) and submicellar cocentrations (CMC/10).

Samples code	Incubation conditions	Inhibition zone diameters (mm)					
		S. aureus ATCC 25923	*S. aureus* MRSA 5578	*E. coli* ATCC 25922	*E. coli* ESBL 135	*C. albicans* ATCC 10231	*C. albicans* CL 2757
PM P84_CURC	470	0	0	0	0	0	0
	darkness	0	0	0	0	0	0
PM P123_CURC	470	0	0	0	0	0	0
	darkness	0	0	0	0	0	0
PM F127_CURC	470	0	0	0	0	0	0
	darkness	0	0	0	0	0	0
Premicellar P84_CURC	470	0	0	0	0	0	0
	darkness	0	0	0	0	0	0
Premicellar P123_CURC	470	0	0	0	0	0	0
	darkness	0	0	0	0	0	0
Premicellar F127_CURC	470	0	0	0	0	0	0
	darkness	0	0	0	0	0	0

Table S2. Minimal inhibitory concentration of tested Pluronic compound at micellar concetrations (1.5% w/v) and submicellar cocentrations (CMC/10).

Samples code	Incubation conditions	Minimal inhibitory concentration (μM/ml)					
		S. aureus ATCC 25923	*S. aureus* MRSA 5578	*E. coli* ATCC 25922	*E. coli* ESBL	*C. albicans* ATCC 10231	*C. albicans* CL 2757
PM P84_CURC	470 nm	>100	>100	>100	>100	>100	>100
	darkness	>100	>100	>100	>100	>100	>100
PM P123_CURC	470 nm	>100	>100	>100	>100	>100	>100
	darkness	>100	>100	>100	>100	>100	>100
PM F127_CURC	470 nm	>100	>100	>100	>100	>100	>100
	darkness	>100	>100	>100	>100	>100	>100
Premicellar P84_CURC	470 nm	>100	>100	>100	>100	>100	>100
	darkness	>100	>100	>100	>100	>100	>100
Premicellar P123_CURC	470 nm	>100	>100	>100	>100	>100	>100
	darkness	>100	>100	>100	>100	>100	>100
Premicellar F127_CURC	470 nm	>100	>100	>100	>100	>100	>100
	darkness	>100	>100	>100	>100	>100	>100
